# Surveillance of Antimicrobial Resistance in Hospital Wastewater: Identification of Carbapenemase-Producing *Klebsiella* spp.

**DOI:** 10.3390/antibiotics11030288

**Published:** 2022-02-22

**Authors:** Miguel Galarde-López, Maria Elena Velazquez-Meza, Miriam Bobadilla-del-Valle, Berta Alicia Carrillo-Quiroz, Patricia Cornejo-Juárez, Alfredo Ponce-de-León, Alejandro Sassoé-González, Celia Mercedes Alpuche-Aranda

**Affiliations:** 1 Centro de Investigación Sobre Enfermedades Infecciosas, Instituto Nacional de Salud Pública, Cuernavaca 62100, Mexico; miguel.galarde@insp.edu.mx (M.G.-L.); berta.carrillo@insp.mx (B.A.C.-Q.); 2 Laboratorio Nacional de Máxima Seguridad para el Estudio de Tuberculosis y Enfermedades Emergentes, Instituto Nacional de Ciencias Médicas y Nutrición “Salvador Zubirán”, Mexico City 14080, Mexico; mbv99@hotmail.com (M.B.-d.-V.); alf.poncedeleon@gmail.com (A.P.-d.-L.); 3 Departamento de Infectología, Instituto Nacional de Cancerología, Mexico City 14080, Mexico; patcornejo@yahoo.com; 4 Unidad de Inteligencia Epidemiológica, Hospital Regional de Alta Especialidad de Ixtapaluca, Ixtapaluca 56530, Mexico; sassoe777@hotmail.com

**Keywords:** *Klebsiella pneumoniae*, carbapenem resistance, waste and treated water, public health

## Abstract

The objective of this study was to investigate the presence and persistence of carbapenemase-producing *Klebsiella* spp. isolated from wastewater and treated wastewater from two tertiary hospitals in Mexico. We conducted a descriptive cross-sectional study in two hospital wastewater treatment plants, which were sampled in February 2020. We obtained 30 *Klebsiella* spp. isolates. Bacterial identification was carried out by the Matrix-Assisted Laser Desorption/Ionization-Time of Flight mass spectrometry (MALDI-TOF MS^®^) and antimicrobial susceptibility profiles were performed using the VITEK2^®^ automated system. The presence of carbapenem resistance genes (CRGs) in *Klebsiella* spp. isolates was confirmed by PCR. Molecular typing was determined by pulsed-field gel electrophoresis (PFGE). High rates of *Klebsiella* spp. resistance to cephalosporins and carbapenems (80%) were observed in isolates from treated wastewater from both hospitals. The molecular screening by PCR showed the presence of *bla_KPC_* and *bla_OXA-48-like_* genes. The PFGE pattern separated the *Klebsiella* isolates into 19 patterns (A–R) with three subtypes (C1, D1, and I1). Microbiological surveillance and identification of resistance genes of clinically important pathogens in hospital wastewater can be a general screening method for early determination of under-detected antimicrobial resistance profiles in hospitals and early warning of outbreaks and difficult-to-treat infections.

## 1. Introduction

The spread of pathogens of public health importance is increasingly rapid between communities and countries [1]. The emerging and re-emerging infectious disease outbreaks have been increasing in recent years, mainly through transmission by direct or indirect contact between humans, animals (including food) and the environment (water) [2,3]. Therefore, an epidemiological surveillance system is an important tool for the identification, monitoring and prediction of infectious diseases [4,5], not only in healthcare institutions, but also through the screening of wastewater environments to detect the presence of resistance bacteria from hospital sources into the community environment [6,7].

Carbapenem-resistant *Enterobacteriaceae* (CRE) are one of the important threats to global public health, generating significant morbidity and mortality as well as associated prolonged healthcare and increased cost [8,9]. CRE are currently defined by the Centers for Disease Control and Prevention (CDC) as: “an isolate of *Enterobacteriaceae* that is resistant to ertapenem, imipenem, meropenem or doripenem according to the Clinical Laboratory Standards Institute (CLSI) or documentation that the isolate produces a carbapenemase” [10,11,12]. There are different bacterial genus belonging to the *Enterobacteriaceae* family, which are present in the microbiota of humans, animals and the environment, among which *Klebsiella pneumoniae* stands out [13,14,15].

The worldwide spread of carbapenemase-producing *Klebsiella pneumoniae* (KPC) is a latent threat due to its capacity for dissemination in healthcare-associated infections (HAIs) and the accumulation of resistance mechanisms through the production of carbapenemase enzymes [16,17,18]. The first strain of KPC was reported in North Carolina in the United States [19], and the geographic distribution was limited to this country until 2005 when KPC was reported in a patient in France [20]. In Latin America, KPC was reported in Colombia in 2006 [21], followed by other countries such as Argentina, Brazil, Uruguay and Chile from 2011–2012 [16]. The prevalence of KPC in Mexico is less than 5%, according to the reports made by Plan Universitario de Control de la Resistencia Antimicrobiana (PUCRA) (2018) and Red Temática de Investigación y Vigilancia de la Farmacorresistencia (INVIFAR) (2020, 2021) [22,23,24]. In Mexico, the first report of KPC-3 (ST258) (n = 24) was made in 2010 as part of an outbreak with a case rate fatality of 55% [25].

KPC strains have been isolated mainly in HAIs in patients with prolonged hospital stays, contaminated medical devices and fomites in healthcare personnel, among others. However, being a ubiquitous bacterium, KPC strains have also been isolated from animals and aquatic environments such as surface water and sewage [13,26,27]. The selection of antibiotic-resistant KPC has increased due to horizontal gene transfer and exchange of mobile genetic elements, producing a great impact on public health [28]. These healthcare-acquired multidrug-resistant bacteria have spread to the environment due to the persistence of antibiotic resistance genes located in mobile genetic elements (MGEs) capable of spreading efficiently among bacteria in the sewage environment [29]. The identification of multidrug-resistance (MDR) or extensively drug-resistance (XDR) bacteria as well as antimicrobial resistant genes in freshwater and wastewater could be considered a good surveillance system for early detection of this event. In addition, the presence and persistence of these bacteria or their material genetics after water treatment could be considered a potentially important medium for the development and spread of antimicrobial resistance, especially in environments with inadequate sanitation [30]. This study explores the presence of carbapenem-resistant *Klebsiella pneumoniae* in hospital wastewater and even after treatment as a means of spread with gradual health risk.

## 2. Results

### 2.1. KPC Isolates

Among the 30 isolates, 26 (86.7%) were confirmed by MALDI-TOF MS^®^ and VITEK 2^®^ as *K. pneumoniae* and 4 (13.3%) as *K. oxytoca*. Half of the isolates were isolated from treated water and the other 50% from raw wastewater. Seven isolates of *K. pneumoniae* (three from raw wastewater/four from treated wastewater) and two of *K. oxytoca* (one from each site) were collected from the wastewater treatment plant (WWTP) of the Hospital Regional de Alta Especialidad de Ixtapaluca (HRAEI), while 19 isolates of *K. pneumoniae* (eleven from raw wastewater/eight from treated wastewater) and two of *K. oxytoca* (both from treated wastewater) were collected at the WWTP of Instituto Nacional de Cancerología (INCAN).

### 2.2. Antimicrobial Susceptibility

The antimicrobial susceptibility results showed that 18 isolates were resistant to ampicillin-sulbactam, ceftazidime and ceftriaxone with a MIC_90_ of >32, >64 and >64 mg/L, respectively, 16 isolates showed resistance to piperacillin-tazobactam, cefoxitin, doripenem, ertapenem and imipenem (MIC_90_ of >128, >64, >8, >8, >16 mg/L, respectively), 17 were resistant to cefepime and ciprofloxacin with a MIC_90_ of >64 and >4 mg/L, eight isolates showed resistance to gentamicin (MIC_90_ of 16 mg/L), only three isolates were resistant to amikacin with a MIC_90_ of 16 mg/L, and all isolates were sensitive to tigecycline with MIC_90_ of 1 mg/L. High rates of *Klebsiella* spp. resistance to cephalosporins and carbapenems (80%) were observed in isolates from treated wastewater from both hospitals.

The isolates collected in both WWTPs were grouped into nine antimicrobial susceptibility profiles. The most frequents profiles were: sensitive to all antibiotics (n = 12) and sensitive only to amikacin, gentamicin and tigecycline (n = 8), (Table 1). Thirteen (81.2%) carbapenem-producing *Klebsiella* spp. were ESBL producers.

### 2.3. Detection of Carbapenem-Resistance Genes

The molecular screening by PCR showed the presence of specific carbapenem-resistance genes (*bla_KPC_* and *bla_OXA-48-like_*); it was observed that 13 (81.25%) *Klebsiella* spp. isolated carried *bla_KPC_* while one isolate was detected to harbor both *bla_KPC_* and *bla_OXA_*_-*48*-*like*_. In only two isolated carbapenem-resistant, the genotype could not be determined; further sequencing analysis is being performed to fully characterized.

### 2.4. Molecular Typing of Pulsed-Field Gel Electrophoresis (PFGE)

**Figure 1:**
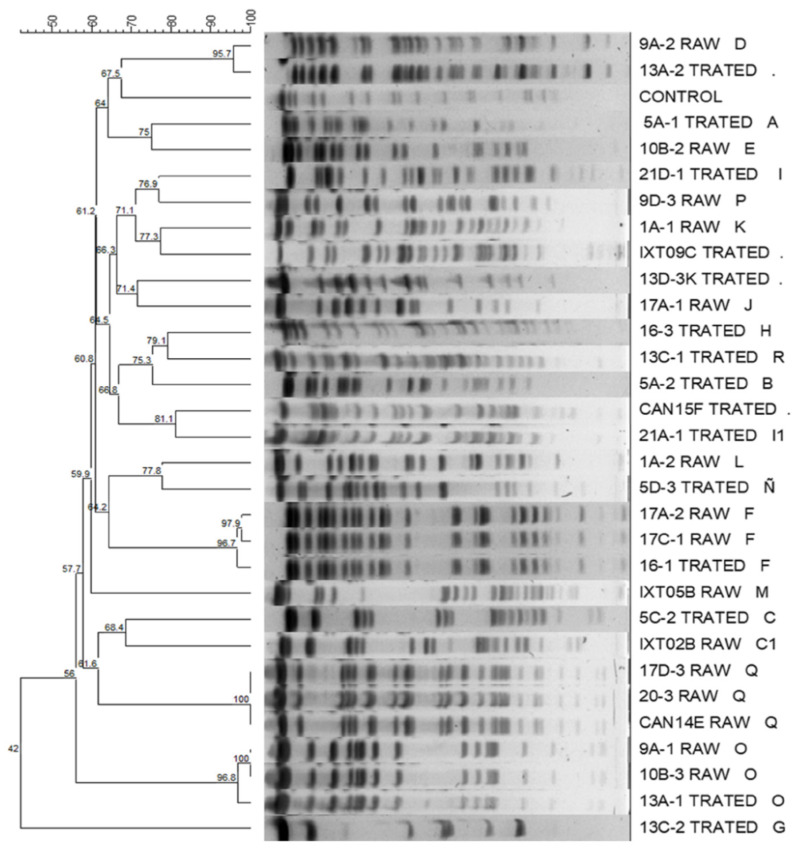
Dendrogram based on pulsed-field gel electrophoresis (PFGE) patterns after digestion with enzyme *Xba* I of *Klebsiella* spp. isolates isolated from hospital wastewater.

The PFGE separated the *Klebsiella* isolates into 19 patterns (A–R) with three subtypes (C1, D1, and I1). The patterns (A, B, C, D1, F, G, H, I, J, N, Ñ, O, R and I1) were present in the *Klebsiella* isolates from the treated wastewater, whereas the patterns (D, E, F, K, L, M, C1, O, P, J and Q) were in the isolates isolated from raw wastewater. The most represented patterns were F, I, O and Q, each with three isolates, and C, D and J, each with two isolates; the remaining patterns were represented by a single isolate. *Klebsiella* isolates with patterns C, D, F, J and O were isolated in samples of raw wastewater and treated wastewater. The clones found were related to the WWTP of origin. It was found that the majority patterns (D, F, I, J, O and Q) were only present in the WWTP of the INCAN, while in the HRAEI WWTP, the *Klebsiella* isolates did not show clonality, except for two isolates (C and C1). The results of the computer analysis of the banding patterns showed similarity percentages that were from 100% to 42% (Figure 1).

When the majority PFGE patterns were compared with the antimicrobial susceptibility profile, it was observed that the isolates with patterns O, Q and J were sensitive to all the antibiotics tested. The isolates with patterns C and F were only sensitive to amikacin, gentamicin and tigecycline; the isolates with pattern I were only sensitive to amikacin and tigecycline, and isolates with pattern D were resistant to all the antibiotics tested, except tigecycline.

## 3. Discussion

HAIs caused by carbapenem-resistant *K. pneumoniae* strains have been reported in hospitals of several regions of the world, including Mexico [8]. However, the present study suggests that hospital wastewater becomes an important source of persistence and spread of multidrug-resistant bacteria, causing these bacteria to escape from WWTPs into the environment. Our findings are important from a public health viewpoint.

Among the total number (30) of *Klebsiella* spp. isolates, 26 were confirmed *K. pneumoniae* and only four as *K. oxytoca;* this is in accordance with a study by Samanta et al., and Sharma et al. also reported the presence of *Klebsiella* spp. in wastewater [31,32]. The present study shows the presence of carbapenemase-producing *Klebsiella* spp. in the studied WWTPs (16/30); some of these isolates were also resistant to beta-lactams (18/30), cephalosporins (17/30), fluoroquinolones (17/30) and aminoglycosides (8/30). In a study conducted in Bangladesh, it was observed that strains of *Klebsiella* spp. (n = 46) isolated from hospital wastewater were resistant to the same groups of antibiotics [26]. In another study carried out in South Africa, 60 *Klebsiella* strains were isolated, of which 28% were resistant to cephalosporins [33], while in Ethiopia in 2012, 30 *Klebsiella* strains were isolated in hospital wastewater, all strains were resistant to ampicillin, ten showed resistance to ceftazidime and six were resistant to gentamicin [27]. These results are similar to those found in our study. In the present study it was observed that 16 (53.3%) isolates of the *Klebsiella* spp. were resistant to carbapenems, similar results were observed in the study by Ebomah et al. [34], where high percentages of resistance to meropenem (69%) were observed. This is contrary to the study by Peneş et al. [35] that reported low resistance to meropenem and imipenem. Some other studies reported *Klebsiella* spp. with a high percentage of resistance against carbapenems as observed in our study for imipenem, carbapenem and meropenem [36,37]. The detection of *K. pneumoniae* strains resistant to carbapenems has also been reported by Perilli et al. [38] in wastewater samples collected throughout a year of study; these strains (n = 20) were also resistant to beta lactams and quinolones, as observed in our work. In addition, it was observed that 80% (12/15) of the *Klebsiella* isolates obtained from treated wastewater were resistant to carbapenems. This result has important implications for public health, since carbapenems are antibiotics of last choice whose resistance may be spreading to the environment through resistant bacteria present in treated wastewater, which is often reused. Interestingly, we found that all the *Klebsiella* spp. isolates analyzed were sensitive to tigecycline; this result is consistent with that reported by Islam et al. [26], who detected that the *K. pneumoniae* strains (n = 46) collected in their study were sensitive to tigecycline; this differs from that reported by Obasi et al. and Teban-Man et al., who detected strains of *K. pneumoniae* resistant to tigecycline isolated from wastewater 57.2 (4/7) and 64% (21/33), respectively [39,40]. The MIC_90_ results in carbapenemase-producing *Klebsiella* spp. isolates in this study ranged from >8 to >16 mg/L, being lower than those reported by Islam et al. [26] in carbapenemase-producing *Klebsiella* spp. isolates obtained from hospital wastewater.

This study detected the presence of CRG in *Klebsiella* isolates and found the genes *bla_KPC_* and *bla_OXA-48-like_*. The OXA-48 gene is one of the most *bla_OXA-48-like_* common variants belonging to class D carbapenemase. The first report of the *bla_OXA-48_* gene was in Turkey in 2001 [18], and the prevalence of OXA-48 genes among *Klebsiella* strains was reported in different countries (Saudi Arabia, China, Turkey and France) [41,42,43,44]. We found that only one strain of *K. pneumoniae* carried *bla_OXA-48-like_*, and this differs from the findings by Gibbon, et al., who reported a high abundance of *bla_OXA-48_* (29%) in strains of *K. pneumoniae* and *K. ornithinolytica* isolated of WWTP influent [45]. The *bla_KPC_* carbapenemases gene has worldwide prevalence [46,47,48]; in our study we detected that 81.25% of the *Klebsiellas* spp. isolates carried the *bla_KPC_* gene, and this is different from the study by Ebomah et al., who reported 10 isolates carried *bla_OXA__-48-like_*; 17 carried *bla_KPC_*; and 73 isolates carried *bla_NDM_*_-*1*_ [34]. As observed in the studies conducted, the frequency of CRG varies in different geographical areas.

Molecular typing by PFGE was performed to investigate the clonal relationship between the *K. pneumoniae* isolates. In our study, we detected a remarkable diversity of patterns (n = 19), but seven patterns predominated; this heterogenicity of patterns and predominance of specific clones has been observed in other studies, such as those published by de Oliveira et al. and Daoud et al., who found three clonal groups and a unique group, respectively, in strains isolated from wastewater [49,50]. Another of our important findings was that the strains of *Klebsiella* spp. that carried the majority of PFGE patterns were found both in raw wastewater and treated wastewater, demonstrating its ability to persist in its passage through the WWTP. The results obtained showed that the majority clonal patterns were related to the WWTP of origin; this could be in relation to the *Klebsiella* clones that circulate in each hospital. The clonality of *K. pneumoniae* in hospitals has been widely documented. In a study carried in Tehran, Iran, PFGE revealed related patterns among *K. pneumoniae* isolates [51]. Another study in Shiraz, Iran, PFGE analysis showed 11 patterns of *K. pneumoniae*, observing important clonal relationships [52].

Although carbapenem-resistant and beta-lactam-resistant enterobacteria are widely explored in the hospital setting, little is known about this resistance phenotype in other environments. There are studies on *Klebsiella* spp. strains resistant to these groups of antimicrobials in different aquatic environments (rivers, sewage, among others) [53,54,55]. Resistance to other antibiotics such as colistin is a point that we propose to address in the carbapenemase-producing *Klebsiella* spp. isolates collected in this study.

These results highlight the need to carry out continuous monitoring programs for antibiotic-resistant bacteria in hospital WWTPs as part of the biosafety program of each institution. A greater number of studies need to be carried out in the hospital WWTPs of our country, to try to understand and reduce the routes of dissemination of resistant bacteria [56].

## 4. Materials and Methods

### 4.1. Study Sites and Sample Collection

The study was conducted at Mexico City Metropolitan Zone in February 2020. Wastewater samples from the Regional High Specialty Hospital of Ixtapaluca (Hospital Regional de Alta Especialidad de Ixtapaluca—HRAEI) and the National Institute of Oncology (Instituto Nacional de Cancerología—INCAN) were the primary sampling sources. These two tertiary-level hospitals were chosen based on several criteria, such as public management, bed capacity, location and the presence of WWTP in their facilities. Wastewater samples were collected in duplicate, before and after passing through each hospital’s wastewater treatment plant (WWTP); the average inlet flow comes from the hospital wards of each of the care centers. The plant configuration begins with a pretreatment process, which consists of removing coarse waste not degradable by conventional biological processes, then dosing the water flow to the aeration and extended aeration process mediated by regulating valves in several chambers. The treatment continues with a clarification and secondary sedimentation stage consisting of chambers, precipitating the thickened sludge. Finally, a disinfection process is carried out by contact with calcium hypochlorite tablets to conclude its passage through granular activated carbon filters, as well as by ultraviolet light (UV) germicidal equipment of the effluent. Selected samples were collected in sterile 1000 mL containers, transported at 4 °C to the Research Center of Infectious Diseases at the National Institute of Public Health (Instituto Nacional de Salud Pública—INSP) for processing.

### 4.2. Microbial Culturing and Identification

Microbial culturing was performed within four hours of sample collection. We used an aliquot of 100 mL of the raw wastewater and 200 mL of the treated wastewater to be centrifuged at 5000 rpm for 20 min; the pellet was dissolved in 10 mL of phosphate buffer solution (PBS). Prior to plating, the samples were diluted 1:1000 in PBS, and then were plating in HiCrome™ ECC Agar plates and then incubated at 37 °C for 18 h. Different presumptive isolates of *Klebsiella* spp. were selected for further identification. A total of 30 isolates of *Klebsiella* spp. were identified by mass spectrometry, using the Microflex LT MALDI-TOF MS^®^ (Bruker Daltonics, Bremen, Germany) equipment.

### 4.3. Antimicrobial Susceptibility Testing

The antimicrobial susceptibility profiles and detection of ESBLs of *Klebsiella* spp. isolates were performed by the VITEK 2^®^ automated system (BioMérieux, Marcy l’Etoile, France). A panel of 14 antimicrobials were used: ampicillin-sulbactam (AMP), piperacillin-tazobactam (TZP), cefoxitin (FOX), ceftazidime (CAZ), ceftriaxone (CTX), cefepime (CEF), doripenem (DOR), ertapenem (ERT), imipenem (IPM), meropenem (MEM), amikacin (AMK), gentamicin (GEN), ciprofloxacin (CIP) and tigecycline (TIG). The minimum inhibitory concentration (MIC) was interpreted according to the breakpoints established by the CLSI, 2021 document [12].

### 4.4. Detection of Carbapenem-Resistance Genes

The presence of carbapenem resistance genes (CRGs) in *Klebsiella* spp. isolates was confirmed by PCR. The assay included the following CRGs: *bla_KPC_, bla_OXA-48-like_, bla_NDM-1_* and *bla_IMP_* [57,58,59,60]. The bacterial DNA was extracted by performing the boiling method, and the DNA templates were stored at −20 °C for PCR analysis. PCR products were visualized by electrophoresis on ethidium bromide stained 1% agarose gel. The primers used, sizes of the amplicons and reaction protocols are listed in Table 2.

### 4.5. Molecular Typing of PFGE

The genomic DNA was prepared as described above; after digestion with *Xba* I endonuclease, the DNA was separated using a CHEF-DR II system (BioRad, Birmingham, UK) [61]. The *Salmonella* serotype Braenderup strain (H9812) was included in each PFGE gel as control. The different clones were compared according to the criteria of Tenover [62].

### 4.6. Computer Fingerprint Analysis

Computer analysis of PFGE profiles was done using the Gel Compar II software, v.6.6.11 (Applied Maths, Inc.; Sint-Martens-Latem, Belgium) after visual inspection. The Lambda PFGE Ladder marker was included in each gel to normalize the PFGE profiles. The Dice coefficients were calculated and were then transformed into an agglomerative cluster by the unweighted pair group method with arithmetic average (UPGMA).

## 5. Conclusions

The results of this work showed the presence of carbapenemase-producing *Klebsiella* spp. isolates in the influents and effluents of the hospital wastewater treatment plants studied, evidencing the ability of these pathogens to survive WWTP treatment systems, as well as their potential ability to persist and spread through wastewater to other environments. The detection of carbapenemase-producing *Klebsiella* spp. isolates in hospital wastewater in our country highlights the need to establish antimicrobial resistance surveillance programs for this and other clinically important microorganisms.

The presence of *Klebsiella* isolates in hospital wastewater may create a real problem for public health, mainly due to the capacity for dissemination of these bacteria and their genes. Given the rapid spread of antimicrobial resistance in different ecological niches around the world, epidemiological surveillance of antimicrobial resistance raises the possibility of monitoring the persistence and spread of antimicrobial-resistant bacteria and their resistance genes not only in the hospital environment, but also in niches where their residues converge, such as wastewater.

## Figures and Tables

**Table 1:** Phenotyping properties of carbapenemase-producing *Klebsiella* spp. present in wastewater treatment plant (WWTP) of Hospital Regional de Alta Especialidad de Ixtapaluca (HRAEI) and Instituto Nacional de Cancerología (INCAN).

Hospital	Sample Site	Code	Isolated	Genes	AMP	TZP	FOX	CAZ	CTX	CEF	DOR	ERT	IPM	MEM	AMK	GEN	CIP	TIG
HRAEI	Raw Wastewater	1A-1	*K. pneumoniae*		16	<4	<4	4	16	<1	<0.12	<0.5	<0.25	<0.25	<2	1	<0.025	<0.5
		1A-2	*K. pneumoniae*		4	<4	<4	<1	<1	<1	<0.12	<0.5	<0.25	<0.25	<2	1	<0.025	<0.5
		IXT02B	*K. pneumoniae*	*bla_KPC_, bla_OXA_*	>32	>128	16	>64	16	2	>8	>8	8	>16	2	1	1	<0.5
		IXT05B	*K. oxytoca*		4	<4	<4	<1	<1	<1	<0.12	<0.5	<0.25	<0.25	<2	1	<0.025	<0.5
	Treated Wastewater	5A-1	*K. pneumoniae*	*bla_KPC_*	>32	>128	16	>64	>64	>64	>8	>8	>16	>16	<2	>16	2	2
		5A-2	*K. pneumoniae*		>32	8	<4	4	>64	2	<0.12	<0.5	<0.25	<0.25	4	<1	2	2
		5C-2	*K. pneumoniae*	*bla_KPC_*	>32	>128	16	64	16	2	>8	>8	8	>16	2	1	2	<0.5
		5D-3	*K. pneumoniae*		>32	>128	>64	>64	>64	>64	>8	>8	>16	>16	>64	16	>4	<0.5
		IXT09C	*K. oxytoca*		>32	>128	>64	>64	>64	>64	>8	>8	>16	>16	16	1	1	<0.5
INCAN	Raw Wastewater	9A-1	*K. pneumoniae*		4	<4	<4	<1	<1	<1	<0.12	<0.5	<0.25	<0.25	<2	1	<0.025	1
		9A-2	*K. pneumoniae*	*bla_KPC_*	>32	>128	16	64	16	2	>8	>8	8	>16	>64	8	1	1
		9D-3	*K. pneumoniae*		4	<4	<4	<1	<1	<1	<0.12	<0.5	<0.25	<0.25	<2	1	<0.025	1
		10B-2	*K. pneumoniae*		2	<4	<4	<1	<1	<1	<0.12	<0.5	<0.25	<0.25	<2	1	<0.025	1
		10B-3	*K. pneumoniae*		4	<4	<4	<1	<1	<1	<0.12	<0.5	<0.25	<0.25	<2	<1	<0.025	1
		17A-1	*K. pneumoniae*		4	<4	<4	<1	<1	<1	<0.12	<0.5	<0.25	<0.25	<2	1	<0.025	<0.5
		17A-2	*K. pneumoniae*	*bla_KPC_*	>32	>128	8	>64	16	2	>8	>8	>16	>16	2	1	1	<0.5
		17C-1	*K. pneumoniae*	*bla_KPC_*	>32	>128	4	>64	8	2	>8	>8	>16	>16	2	1	1	<0.5
		17D-3	*K. pneumoniae*		4	<4	<4	<1	<1	<1	<0.12	<0.5	<0.25	<0.25	<2	1	<0.025	<0.5
		20-Mar	*K. pneumoniae*		4	<4	<4	<1	<1	<1	<0.12	<0.5	<0.25	<0.25	<2	1	<0.025	<0.5
		CAN14E	*K. pneumoniae*		4	<4	<4	<1	<1	<1	<0.12	<0.5	<0.25	<0.25	<2	1	<0.025	<0.5
	Treated Wastewater	13A-1	*K. pneumoniae*		4	<4	<4	<1	<1	<1	<0.12	<0.5	<0.25	<0.25	<2	<1	<0.025	1
		13A-2	*K. pneumoniae*	*bla_KPC_*	>32	>128	16	>64	16	2	>8	>8	>16	>16	>64	8	0.5	1
		13C-1	*K. oxytoca*	*bla_KPC_*	>32	>128	4	>64	16	2	>8	>8	>16	>16	2	1	>4	<0.5
		13C-2	*K. pneumoniae*	*bla_KPC_*	>32	>128	>64	>64	32	8	>8	>8	8	>16	<2	8	1	1
		16-ene	*K. pneumoniae*	*bla_KPC_*	>32	>128	8	>64	>64	2	>8	>8	8	>16	2	1	1	<0.5
		16-Mar	*K. oxytoca*	*bla_KPC_*	>32	>128	>64	>64	>64	32	>8	>8	>16	>16	8	1	2	2
		21A-1	*K. pneumoniae*	*bla_KPC_*	>32	>128	8	>64	8	2	>8	4	8	>16	2	8	2	1
		21D-1	*K. pneumoniae*	*bla_KPC_*	>32	>128	8	>64	16	2	>8	>8	>16	>16	2	>16	>4	1
		CAN15F	*K. pneumoniae*	*bla_KPC_*	>32	>128	8	64	8	2	>8	4	8	>16	<2	>16	>4	1
		13D-3-k	*K. pneumoniae*		4	<4	<4	<1	<1	<1	<0.12	<0.5	<0.25	<0.25	<2	1	<0.025	1

Ampicillin-sulbactam (AMP), piperacillin-tazobactam (TZP), cefoxitin (FOX), ceftazidime (CAZ), ceftriaxone (CTX), cefepime (CEF), doripenem (DOR), ertapenem (ERT), imipenem (IPM), meropenem (MEM), amikacin (AMK), gentamicin (GEN), ciprofloxacin (CIP) and tigecycline (TIG).

**Table 2:** Primer sequences of target carbapenems genes and their respective amplicon sizes and PCR cycling conditions.

Target Gene	Primer Sequence (5’-3’)	Amplicon Size (bp)	PCR Cycling Condition	References
*bla_NDM-1_*	F: GGTTTGGCGATCTGGTTTTC R: GGAATGGCTCATCACGATC	621	Initial denaturation 10 min at 94 °C. Denaturation at 94 °C for 30 s, annealing at 52 °C for 40 s, extension at 72 °C for 50 s, by 36 cycles, and final extension at 72 °C for 10 min.	Dogonchi AA, et al. [57]
*bla_IMP_*	F: ATAGCCATCCTTGTTTAGCTC R: TCTGCGATTACTTTATCCTC	818	Initial denaturation 10 min at 94 °C. Denaturation at 94 °C for 30 s, annealing at 58 °C for 30 s, extension at 72 °C for 1 min by 30 cycles, and final extension at 72 °C for 10 min.	Aubron, CL, et al. [58]
*bla_KPC_*	F: ATGTCACTGTATCGCCGTCT R: TTTTCAGAGCCTTACTGCCC	893	Initial denaturation 15 min at 95 °C. Denaturation at 94 °C for 1 min, annealing at 62 °C for 1 min, extension at 72 °C for 1 min by 38 cycles, and final extension at 72 °C for 10 min.	Bradford, PA, et al. [59]
*bla_OXA-48-like_*	F: TTGGTGGCATCGATTATCGG R: GAGCACTTCTTTTGTGATGGC	744	Initial denaturation 5 min at 94 °C. Denaturation at 94 °C for 1 min, annealing at 60 °C for 1 min, extension at 72 °C for 1 min by 30 cycles, and final extension at 72 °C for 10 min.	Poirel, LC, et al. [60]

## Data Availability

Not applicable.
